# Changes in the Bacterial Community of Soil from a Neutral Mine Drainage Channel

**DOI:** 10.1371/journal.pone.0096605

**Published:** 2014-05-05

**Authors:** Letícia Bianca Pereira, Renato Vicentini, Laura M. M. Ottoboni

**Affiliations:** Center for Molecular Biology and Genetic Engineering (CBMEG), State University of Campinas – UNICAMP, Campinas, SP, Brazil; American University in Cairo, Egypt

## Abstract

Mine drainage is an important environmental disturbance that affects the chemical and biological components in natural resources. However, little is known about the effects of neutral mine drainage on the soil bacteria community. Here, a high-throughput 16S rDNA pyrosequencing approach was used to evaluate differences in composition, structure, and diversity of bacteria communities in samples from a neutral drainage channel, and soil next to the channel, at the Sossego copper mine in Brazil. Advanced statistical analyses were used to explore the relationships between the biological and chemical data. The results showed that the neutral mine drainage caused changes in the composition and structure of the microbial community, but not in its diversity. The *Deinococcus/Thermus* phylum, especially the *Meiothermus* genus, was in large part responsible for the differences between the communities, and was positively associated with the presence of copper and other heavy metals in the environmental samples. Other important parameters that influenced the bacterial diversity and composition were the elements potassium, sodium, nickel, and zinc, as well as pH. The findings contribute to the understanding of bacterial diversity in soils impacted by neutral mine drainage, and demonstrate that heavy metals play an important role in shaping the microbial population in mine environments.

## Introduction

The mining of metal ores and coal can lead to a variety of environmental problems, including deforestation, soil erosion, and the flooding of low-lying areas. One of the most critical issues in mine environments is the natural oxidation (chemical and biological) of sulfide mineral tailings that are exposed to water, oxygen, and microorganisms. This oxidation is responsible for the generation of mine drainage that compromises the quality of soil, surface water, and sub-surface water bodies, hence affecting overall biodiversity [1], [2].

Mine drainage can be highly acidic or alkaline, depending on the complex interactions of hydrological, chemical, and biological processes. In addition, mine drainages are rich in heavy metals and sulfur, which are present in the minerals, but poor in nutrients. As a result, mine drainage is a multi-factor pollutant (considering aspects such as acidity or alkalinity, salinization, metal toxicity, and sedimentation processes), with the importance of each factor depending on the characteristics of the environment affected [2], [3], [4].

Investigation of the microbial communities of these environments can reveal undiscovered species able to provide a pool of genes and proteins whose potential is still unknown. Microorganisms in mine drainages have been studied for purposes including the bioremediation of mine sites [5] and the development of bioleaching consortia [6], [7]. In addition, the metabolic diversity of the microbial community present in mine drainage makes these environments ideal for studies of genomes, ecology, evolution, tolerance mechanisms, and the interactions between bacteria and environmental factors [8], [9], [10].

Due to the extreme pH conditions (<3.0) and the high content of heavy metals, the microbial community of acid mine drainage (AMD) has been extensively studied. Evaluations have been made of the microbial communities in acid mine drainage [11], [12], river sediments contaminated with AMD [13], subsurface mine environments [14], and AMD sediments [11].

In many cases, due to the neutralizing capacity of the waste minerals, or human intervention such as the spreading of limestone to precipitate metals, the drainage can have higher pH values (4.5 to 8.5), and is then called neutral mine drainage (NMD). This can cause severe environmental problems in mine environments because heavy metals, which are often present at high concentrations, can remain soluble at alkaline pH under suitable redox conditions [1], [15].

Previous studies have reported that NMD contains great bacterial diversity, while that of AMD is typically low. Using metagenomic and metaproteomic approaches, Halter *et al.* [16] found a wide range of different bacteria in slightly alkaline French mine sediments, including iron-oxidizing and heterotrophic organisms. Reis *et al*. [17] reported a high diversity index for alkaline river sediments contaminated with heavy metals released from a Brazilian arsenic mine. However, NMD is an environment that has been poorly explored, and little is known about the structure and diversity of the bacterial community in soil contaminated with this kind of drainage [18], [19].

The interaction between the bacterial community and a heavy metal-contaminated soil is also incompletely understood. Guo *et al*. [19] showed that the heavy metal-contaminated soils of two abandoned copper mines in Australia did not have any impact on microbial diversity. However, Marcin *et al*. [20] reported that a high content of heavy metals in forest soils had a negative influence on bacterial activity and diversity. It is noteworthy that the structure and diversity of bacteria communities in soil is not only influenced by heavy metal concentrations, but also by season [21], pH [22], organic matter [23], and interactions between these factors.

The behavior of the soil bacterial community exposed to different contaminants in extreme environments can be characterized using a combination of high-throughput sequencing, knowledge of the soil chemical parameters, and advanced statistical analysis. In this work, intensive parallel pyrosequencing of 16S rDNA was used to evaluate the microbial community in soil impacted by neutral mine drainage. The influence of soil chemical parameters on the composition, structure, and diversity of the bacterial community was also evaluated.

## Materials and Methods

### Sample collection and chemical analysis

Samples were collected at the Sossego copper mine (6°25'45"S, 50°3'58"W), in Canaã dos Carajás, Brazil. The mine has been operated by Vale since 1997 and the collection was authorized by this Company. In the mine, a drainage flows continuously from an ore deposit along a channel in the soil that starts at an ore heap and terminates at a runoff area. Along the course of the channel, the drainage is treated with limestone to increase the pH and precipitate heavy metals. Six drainage samples (D1-6, see Table S1 for coordinates) were collected aseptically from the upper soil layer (0–10 cm). Six uncontaminated soil samples (S1-6, see Table S1 for coordinates) were also collected (at 0–10 cm), adjacent to each drainage sample. The samples were stored at −20°C.

The chemical parameters Cd, Ca, Pb, Cu, Cr, S, Fe, P, Mg, Mn, Ni, K, Na, and Zn were measured in each sample prepared according to US EPA SW-846 method 3051A, with detection using inductively coupled plasma atomic emission spectrometry (ICP-AES) [24]. The organic matter (OM) content and pH (soil/water  = 1∶2.5, w/v) were determined as described by Camargo *et al*. [25].

### DNA isolation, 16S rDNA library construction, and pyrosequencing

Prior to the isolation of DNA, the heavy metals were removed from the environmental samples using the method of Sánchez-Andrea *et al*. [13], with modifications. For this, the samples (0.5 g) were resuspended in 1 mL of phosphate-buffered saline (PBS), agitated, and centrifuged at 12879 x g for 10 min. The supernatant was discarded and the samples were resuspended in 1 mL of 0.5 M EDTA, at pH 8, and incubated at 4 °C for 2 h. After the incubation, the samples were centrifuged at 12879 x g for 10 min, and the supernatant was discarded. The wash with EDTA was repeated until the metals had been completely removed. The material was then washed with PBS, and the DNA was isolated from a 0.25 g aliquot using the Power Soil DNA Isolation Kit (MoBio Laboratories, Carlsbad, USA), according to the manufacturer's instructions.

The amplification of the V3–V4 region of the 16S rDNA was performed using the primers 338F and 806R [26], [27]. The reaction mixture (final volume 25 µL) consisted of 3–10 ng of DNA, 0.6 µM of each primer, 0.5 U of AccuPrime Pfx DNA Polymerase (Invitrogen), and AccuPrime Pfx 1X reaction mix (Invitrogen). Amplification was performed under the following conditions: initial denaturation at 94°C for 1 min, 20 cycles at 94°C for 15 s, 52°C for 30 s, and 72°C for 30 s, and a final extension at 72°C for 2 min. After the first amplification, a further five cycles were performed, with a final volume of 50 µL and using the same conditions, in order to add specific barcodes to each sample and the adaptors A and B. The purification of the amplicons was accomplished using the GFX PCR DNA and Gel Band Purification Kit (GE Healthcare). An equimolar mixture of the 12 samples was made in order to obtain 2400 ng of PCR product with a concentration of 16.48 ng/µL. The amplicons were sequenced on a 454 GS Junior platform (Roche Company, Branford, CT, USA). The pyrosequencing data were submitted to MG-RAST [28] (ID 4521082.3 to 4521090.3 and 4521341.3 to 4521343.3) and to NIH Sequence Read Archive (BioProject PRJNA239576, accession numbers SRR1178540 and SRR1179196).

### Sequence processing and statistical analysis

The QIIME package [29] was used to analyze the quality of the sequences and to group them into operational taxonomic units (OTUs, similarity of 97% or greater). Sequences with a quality score ≥ 25 and size between 400 and 470 bp were used in the analysis. Ambiguous bases and mismatches in primer sequences were not admitted. Chimeras were checked and removed with the ChimeraSlayer algorithm in QIIME. The taxonomic classification was performed using the RDP (Ribosomal Database Project, http://rdp.cme.msu.edu/classifier/classifier.jsp) at the 80% threshold.

The Student's t-test was employed to compare the drainage and soil samples in terms of the chemical parameters, using Statistica v.10 software [30]. Analysis of similarities (ANOSIM) was used to test the significance of the differences observed between the drainage and soil samples, based on the Bray-Curtis distance, considering the OTU composition of the samples. The t-test (with 95% confidence intervals) was used to determine whether the means of the Simpson, Shannon, and Berger-Parker diversity indices were statistically different, in terms of species richness and dominance, between the drainage and soil samples. Non-metric multidimensional scaling (n-MDS) was carried out using the Bray-Curtis distance matrix to plot the distances between the samples, considering the OTU distribution and the chemical parameters. Similarity percentage (SIMPER) analysis was used to identify the taxa that were mainly responsible for the differences observed between the drainage and soil samples. All the analyses were performed with normalized data, by sample length, using the PAST software [31].

Multivariate regression tree (MRT), aggregated boosted tree (ABT), and Spearman rank correlation analyses were carried out to correlate the chemical parameters with the biological data. MRT [32] was used to explore the relationship of the chemical parameters with the relative abundance of the dominant phyla and standardized diversity indices. This analysis was performed using the mvpart package within the R statistical environment, with default parameters. ABT [33] was performed (with 5000 trees, 10-fold cross validation, and three-way interaction) to evaluate the relative importance of the chemical parameters to the relative abundance of the dominant phyla, using the gbmplus package within the R environment. Non-parametric Spearman rank correlation was used to test the significance (p<0.5) of the relationships between the chemical parameters and phyla abundance, using the Statistica v.10 software.

## Results

### Chemical characterization of samples

A total of 16 chemical parameters were examined for each sample. Significant differences between the drainage and soil samples were observed for copper, nickel, potassium, sodium, and zinc (Table 1). The concentrations of the heavy metals were higher in the drainage samples, while those of sodium and potassium were higher in the soils. The pH of the soil samples ranged from 4.86 to 8.66, while pH values between 6.08 and 8.05 indicated that the drainage was a neutral mine drainage. Also, high concentrations of calcium were observed in samples S3, S5, and S6. This was due to the addition of limestone along the drainage channel of the Sossego mine, which also increased the pH and the calcium concentration of the soil next to the drainage (see Table S1 for details). High sulfur concentrations were observed in samples D3 and S2, which can be explained by the fact that the drainage channel flows over uneven ground, resulting in the accumulation of certain elements in some of the drainage and nearby soil samples.

**Table 1:** Chemical parameters of the drainage and soil samples, and P-values derived from the Student's t-test.

	Drainage Mean values	Soil Mean values	P-value
**Cadmium**	0.60±0.15	0.47±0.14	0.15
**Calcium**	40266.67±74148.52	28633.33±50360.92	0.76
**Lead**	5.35±2.86	5.85±4.49	0.82
**Copper**	31436.33±22707.81	6287.17±8012.76	0.03*
**Chromium**	22.75±10.29	26.45±9.86	0.54
**Sulfur**	24391.67±53721.94	28865.00±69631.71	0.90
**Iron**	27603.50±15210.08	21695.83±9405.91	0.43
**Phosphorus**	11286.67±23115.07	538.33±253.57	0.28
**Magnesium**	7608.33±4871.39	4883.33±4937.37	0.36
**Manganese**	324.83±139.01	446.50±413.48	0.51
**Nickel**	112.25±42.2	64.95±26.24	0.04*
**Potassium**	9.32±8.44	268.50±157.6	0.002**
**Sodium**	1.82±0.91	102.35±45.13	0.00**
**Zinc**	31.17±18.34	13.22±3.13	0.04*
**pH**	7.50±0.71	7.25±1.59	0.73
**OM**	22.50±9.12	22.33±17.22	0.98

Metal concentrations are expressed in mg/kg. The organic matter (OM) content is expressed in g/dm^3^.

* Statistically significant at 5%.

** Statistically significant at 1%.

### Taxonomic composition

The pyrosequencing resulted in 102655 quality sequences from the 12 samples analyzed. The number of sequences per sample ranged from 3248 to 21198 (8554.58±5390.96). A total of 5901 OTUs were found, and approximately 45% were singletons. Of these OTUs, 5636 (95.5%) could be assigned to at least one particular phylum, and 24 OTUs were assigned to *Archea*. The Good's coverage ratio of the samples ranged from 83.8 to 99% (93.22±4.67%) (see Table S2 for details).

**Figure 1:**
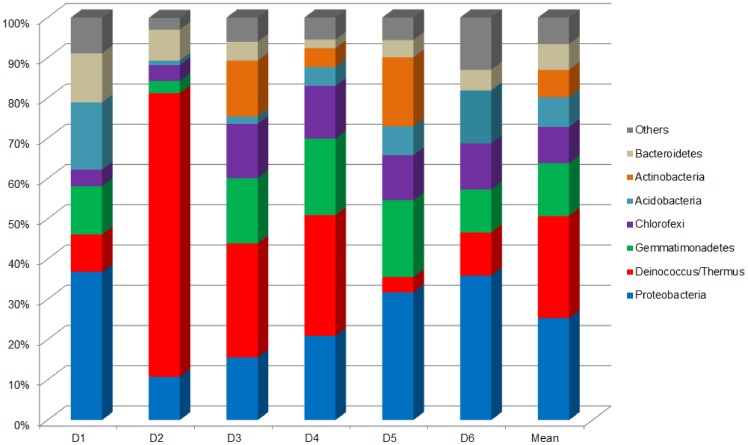
Relative abundance of bacterial phyla in drainage samples. Others: Armatimonadetes, BRC1, Chlamydiae, Chlorobi, Cyanobacteria, Elusimicrobia, Firmicutes, Nitrospirae, OD1, Planctomycetes, Spirochaetes, Synergistetes, Verrucomicrobia, TM6, TM7, WPS-2, WS3, and unclassified Bacteria.

**Figure 2:**
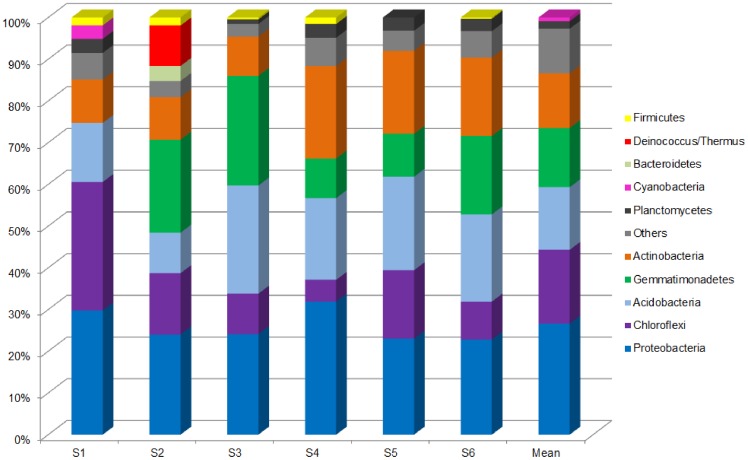
Relative abundance of bacterial phyla in soil samples. Others: Acidobacteria, AD3, Armatimonadetes, Chlamydiae, Chlorobi, Elusimicrobia, Fusobacteria, GAL15 Nitrospirae, OD1, Spirochaetes, Tenericutes, Verrucomicrobia, TM6, TM7, WPS-2, WS3, WYO, and unclassified Bacteria.

The phyla *Proteobacteria* (25.3%) and *Deinococcus/Thermus* (25.4%) were dominant in the drainage samples (Figure 1), while in soil the dominant phyla were *Proteobacteria* (26.6%), *Chloroflexi* (17.7%), *Acidobacteria* (15%), *Gemmatimonadetes* (14.2%), and *Actinobacteria* (13.1%) (Figure 2). Among the *Proteobacteria,* the most dominant taxa in both environments were *Alphaproteobacteria* (44.7% in drainage and 51.7% in soil), followed by *Betaproteobacteria* (26.5% in drainage and 19% in soil). In general, variation of the relative abundance of different phyla was more notable in the soil samples, while the drainage environment was dominated by a few phyla.

The drainage samples presented a total of 3023 OTUs, while the soil samples presented 4031 OTUs. Only 1153 OTUs (19.5%) were shared between both environments; most OTUs were found exclusively in one of the environments analyzed. All these results suggest that the composition of the bacterial community differed between the mine drainage and soil environments.

### Diversity analysis

The analysis of similarities (ANOSIM), which considered the OTU compositions, revealed significant differences between the drainage and soil bacterial communities (p = 0.0087). No significant difference was found when the drainage samples were compared to each other (p = 1), and the same was observed for the soil samples.

**Figure 3:**
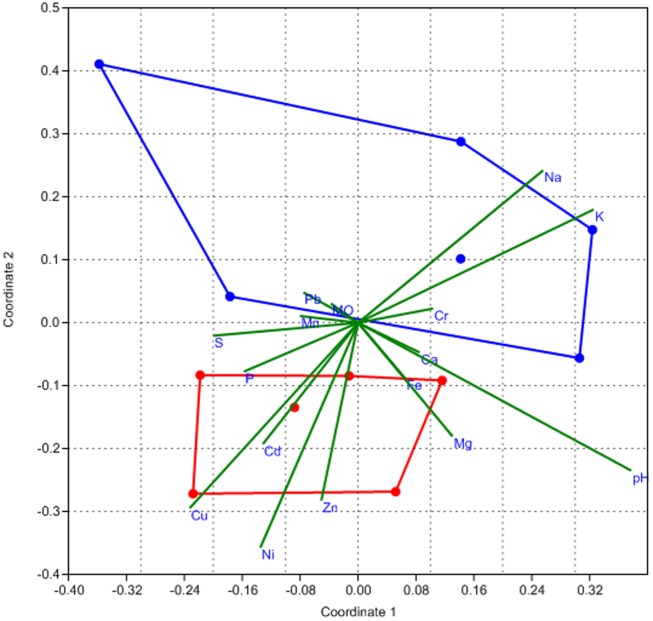
Non-metric multidimensional scaling. Non-metric multidimensional scaling using the Bray-Curtis dissimilarity plots of the first two components for drainage (red) and soil (blue) samples. The stress value is 0.095.

Non-metric multidimensional scaling (n-MDS) (Figure 3) was performed to group the samples by associating the chemical parameters and the OTU frequencies. The results indicated a clear separation between the drainage and soil samples. The soil samples showed more scatter, suggesting greater heterogeneity, and the concentrations of sodium and potassium were the chemical parameters that contributed most to the separation. The drainage samples showed a more homogenous distribution, closely related to the amounts of copper, cadmium, nickel, and zinc. These results were in accordance with the statistical analysis (Student's t-test) showing that the amounts of the chemical parameters described above were different for the two environments. The ANOSIM and n-MDS analyses indicated that the structure of the bacterial community changed when the environment was directly impacted by the mine drainage.

The Student's t-test applied to the diversity indices revealed that, considering all the samples, there were no significant differences between the drainage and soil in terms of diversity (p = 0.210, Shannon index) and dominance (p = 0.26, Berger-Parker index; p = 0.25, Simpson index).

Since a difference in the bacterial community was observed between the environments, SIMPER analysis was used to show which OTUs contributed to this result. The five main OTUs are shown in Table 2. The OTU that contributed the most to the differences between the soil and drainage communities was 824 (12.7% of the total dissimilarity), composed of the genus *Meiothermus*, which was highly abundant in the drainage samples. The OTUs 3607 and 4974 were classified as *Gemmatimonas*, and were more abundant in the soil samples. Together, these OTUs accounted for 4.43% of the difference. The OTU 1366 was identified as *Acidobacteria*, a phylum containing genera commonly found in soil and drainage [14]. The OTU 303 was classified as *Bacteria*, and presented 95% identity with soil bacteria. The high contribution of OTU 824 is particularly interesting, because it was the dominant OTU in all the drainage samples and was most important in the species dominance index (Berger-Parker index, data not shown). These results revealed that the presence of the mine drainage had a positive influence on the abundance of this OTU in the environment.

**Table 2:** Principal OTUs responsible for differences between the drainage and soil samples.

OTU	Dissimilarity contribution (%)	Abundance Drainage (%)	Abundance Soil (%)	RDP classifier [bootstrap value]
**824**	12.7	23.3	1.09	*Meiothermus* [99%]
**3607**	2.69	2.5	3.31	*Gemmatimonas* [99%]
**303**	1.8	0.19	3.11	*Bacteria* [100%]
**1366**	1.78	3.45	0.37	*Acidobacteria* [100%]
**4974**	1.74	0.012	3.06	*Gemmatimonas* [89%]

### Influence of the chemical parameters on microbial composition and diversity

**Figure 4:**
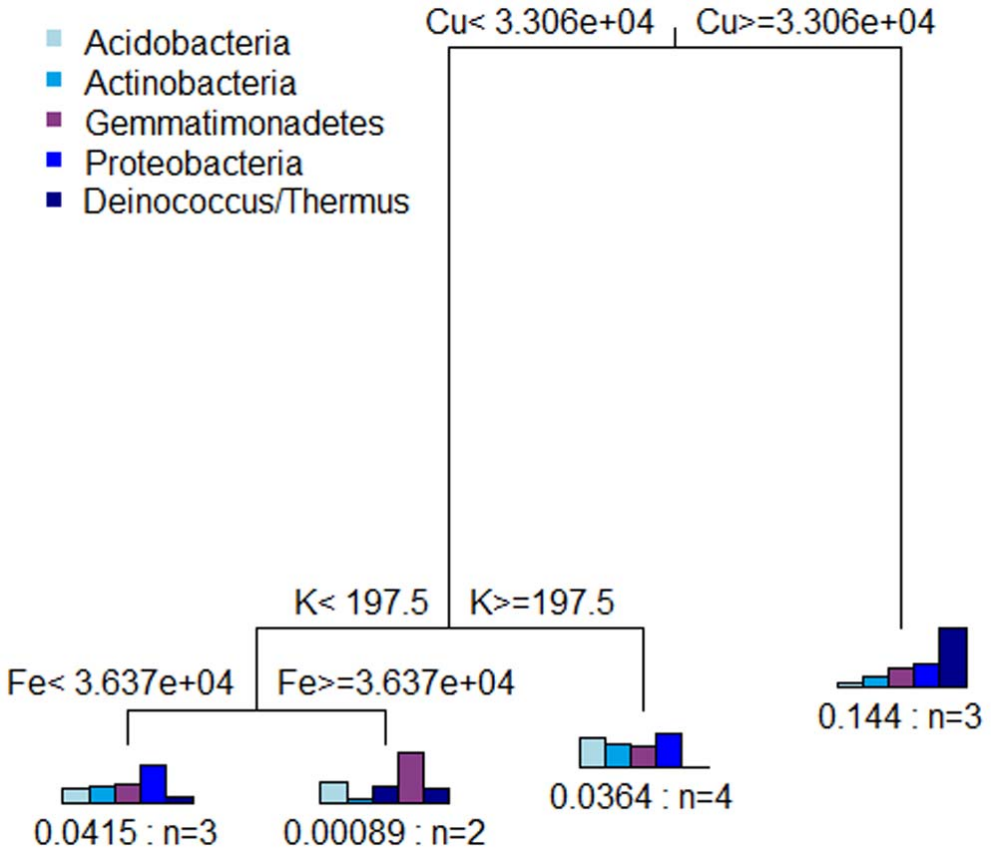
Multivariate regression tree of the abundance of phylum. Multivariate regression tree of the relation between relative abundance of dominant phylum and chemical parameters. The bar plots show the mean relative abundance of each phylum at the terminal nodes. The numbers under the bar plots indicate the error and the number of samples (n) within each group. Error: 0.299.

The MRT analysis was conducted by associating the chemical parameters with both the relative phylum abundance and the standardized diversity indices. For the distribution of the dominant phylum, the environmental data provided a tree with three splits, based on copper, potassium, and iron (Figure 4). The tree explained 70% of the variance of the relative phylum abundance. In this tree, copper was responsible for the first split, appearing to be a strong predictor in this environment since it explained 58.4% of the variance. *Deinococcus/Thermus* were the most abundant phyla in samples exhibiting a high content of copper, while other phyla were less abundant.

The second and third splits were determined by potassium (8.2% of the variance) and iron (3.5% of the variance), respectively. *Proteobacteria* was abundant in samples that contained a smaller amount of potassium and were richer in iron. In contrast, *Acidobacteria* and *Actinobacteria* were prevalent in samples containing higher amounts of potassium, and the *Actinobacteria* population decreased when the amount of iron increased. *Gemmatimonadetes* showed a homogeneous distribution along the tree.

**Figure 5:**
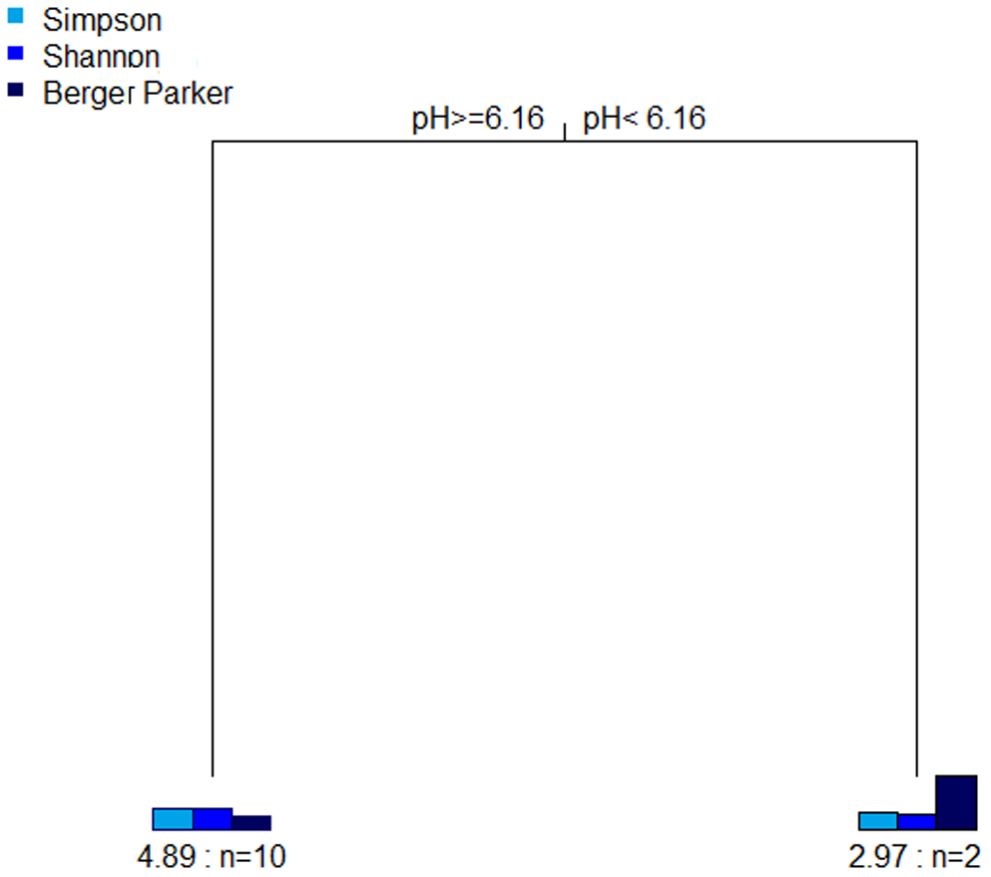
Multivariate regression tree of the diversity indices. Multivariate regression tree of the relation between standardized diversity indices and chemical parameters. The bar plots show the mean of the diversity indices at the terminal nodes. The numbers under the bar plots indicate the error and the number of samples (n) within each group. Error: 0.744.

The tree for the standardized diversity indices (Figure 5) showed one split determined by pH. This tree explained 58.1% of the variance. As shown by the split, the Berger-Parker index decreased at pH above 6.16, while the Shannon and Simpson indices showed higher values, suggesting less dominance and greater diversity in the alkaline environments. Additionally, the single regression tree with the diversity indices (Figure S1) showed that the Shannon index was explained more by potassium and pH, while the Berger-Parker and Simpson indices were explained by pH.

**Figure 6:**
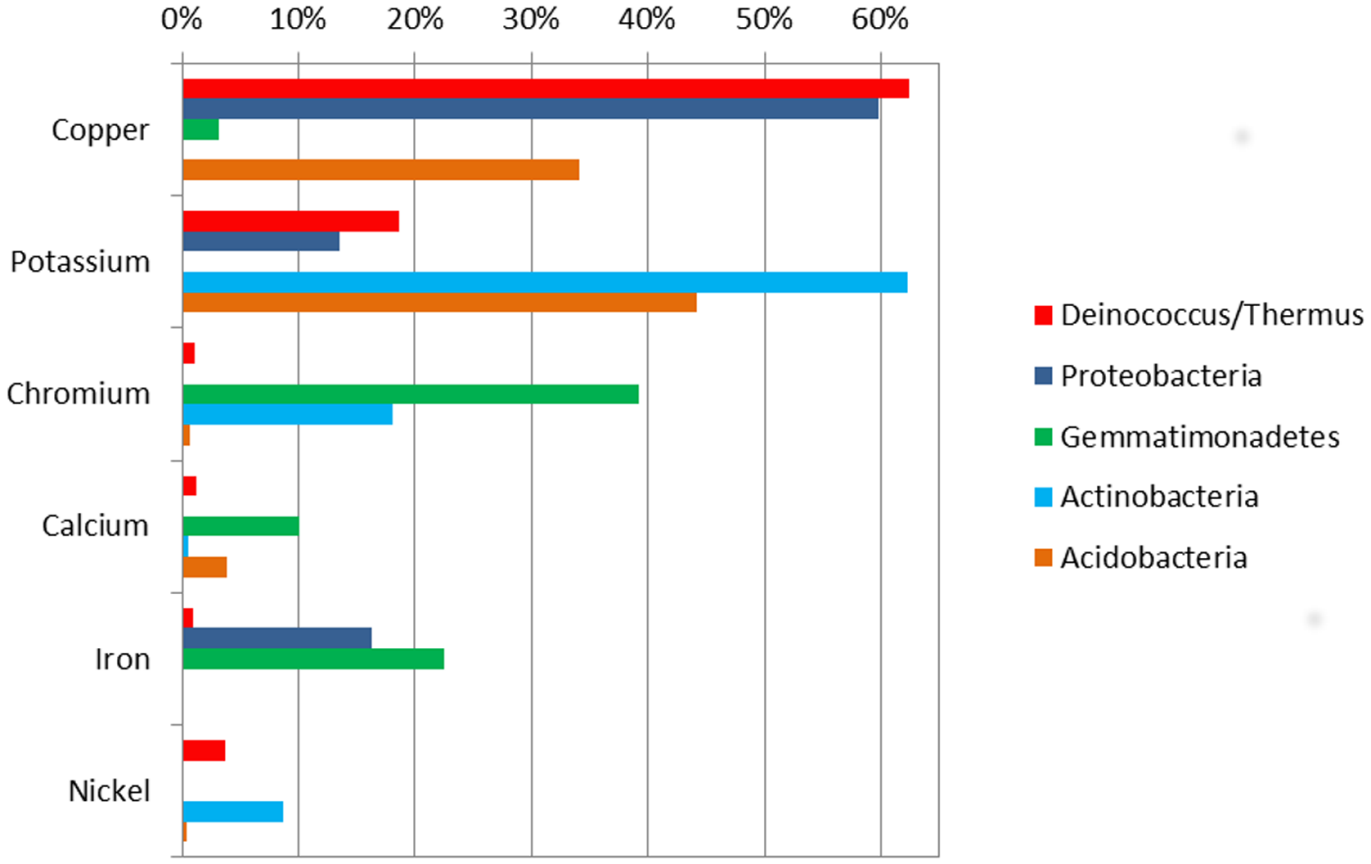
Aggregated boosted tree (ABT). The ABT analysis show the relative influence of the principal chemical parameters for the dominant phyla in the Sossego mine.

The ABT models showed how the relative importance of the chemical parameters influenced the relative abundance of the dominant phyla (Figure 6). In general, the analysis indicated that copper and potassium were the principal parameters that exerted a strong influence on these phyla. Copper was the principal influence on *Proteobacteria* (59.74%) and *Deinococcus/Thermus* (62.42%), and an intermediate influence on *Acidobacteria* (34.16%) and *Gemmatimonadetes* (3.12%). This metal showed no influence on *Actinobacteria*. Potassium was the principal influence on the distribution of the *Acidobacteria* (44.14%) and *Actinobacteria* (62.24%) communities, and showed an intermediate influence on *Proteobacteria* (13.58%) and *Deinococcus/Thermus* (18.65%). These findings supported the MRT results, showing the importance of these chemical parameters in shaping the microbial communities in the mine environments. Chromium, calcium, iron, and nickel were also important parameters that influenced the distribution of the principal phyla (see Table S3 for complete relative influence data).

Spearman rank correlation analysis (Table S4) revealed a slightly positive correlation between *Acidobacteria* and *Actinobacteria/Proteobacteria*. *Gemmatimonadetes* presented significant correlations with cadmium, chromium, and sulfur, while *Proteobacteria* was negatively correlated with calcium and pH. The *Thermus* phyla was highly positively correlated with copper, potassium, and zinc, and slightly correlated with sulfur, iron, and nickel, reinforcing the previous findings for the relationships between the chemical and biological data.

## Discussion

This work evaluated the impact of a neutral mine drainage flow on the soil microbial community. Statistical analysis of the measured chemical parameters revealed significantly higher levels of copper, nickel, and zinc in the drainage samples, compared to the soil samples. The increase of these metals in the drainage was expected because they were part of the composition of the minerals in the Sossego mine ore deposit, which after oxidation became soluble and flowed along the soil. In contrast, the soil samples contained higher amounts of sodium and potassium. These chemical components could have played an important role in shaping the microbial community in these environments. However, it is important to note that both the drainage and soil samples presented high contents of heavy metals, iron, and sulfur, in addition to the calcium and magnesium that increase the normal pH of the soil. Therefore, both environments (soil and drainage) presented conditions that were adverse to microbial communities, and deserved to be investigated.

Analysis of microbial composition revealed that the bacterial community in the drainage samples was dominated by only a few phyla, with only two phyla (*Proteobacteria* and *Deinococcus/Thermus*) accounting for 50% of the total diversity. The microbial community of the soil samples presented more phyla with similar relative abundances. In areas contaminated with heavy metals, it has been observed that the abundance of tolerant bacteria increases, while that of the more sensitive organisms decreases [34]. Therefore, the higher content of metals such as copper, nickel, and zinc in the drainage samples could have acted to select the resistant bacteria that dominated this environment. *Proteobacteria* was the phylum with the highest relative abundance in both drainage and soil samples. *Proteobacteria* has been found to be the predominant phylum in many mine environments, including arsenic mine sediment [16], gold mine stream sediments [17], and a river contaminated with metals [35]. This phylum exhibits a complex lifestyle and can degrade a variety of complex organic molecules, enabling it to adapt to many different environments [23].

The small number of OTUs shared between samples supports the hypothesis of the existence of large differences between the bacterial community compositions of the drainage and soil samples. The n-MDS and ANOSIM analyses showed that there were differences between the two environments in terms of the structure of the communities. The high heterogeneity observed among the soil samples reflected the wide variety of phyla in this environment, as observed in the community composition results. In contrast, the drainage samples exhibited less diversity, suggesting that in this environment, the bacterial community was more homogeneous because fewer organisms could adapt to the extreme conditions.

Comparison of the Simpson, Shannon, and Berger-Parker indices revealed that there was no significant difference between the drainage and soil samples in terms of diversity. According to Kandeler *et al*. [36], enrichment of the soil with heavy metals reduces the biomass and activity of bacteria. However, recent studies have shown that after a century of exposure to acid drainage, the bacterial community tends to stabilize and increase its diversity, resulting in an environment with a microbial diversity similar to that of non-impacted areas [17], [23]. The Sossego mine has been in operation for 16 years, and soil contamination is considered to be relatively recent. Therefore, it was expected that diversity would be lower at the drainage sites because only a few groups of bacteria would be resistant to the extreme conditions of this environment. However, it has been suggested that while acute exposure has a negative influence on bacterial diversity, in chronically contaminated environments, the proliferation of resistant bacteria can increase diversity [37], [38]. This situation is common in mines such as the Sossego mine, where the drainage flows constantly from the ore deposit. Furthermore, high concentrations of nutrients can improve diversity in sediments contaminated with heavy metals [17], [39]. At the Sossego mine, the drainage flows over the soil to a run-off area located about 700 m from the ore deposit, and the vegetation in this area provides high levels of organic matter. The natural nutrient content of this soil might therefore play a key role in the promotion of bacterial diversity.

The SIMPER results revealed that OTU 824, associated with *Meiothermus*, accounted for much of the difference between the drainage and soil communities, because it was much more abundant in the drainage samples. The optimum growth temperatures of these bacteria lie between 50 and 65°C, optimum pH is around 8.0, and they are usually found in warm and nutrient-poor environments, such as geothermal and anthropogenically-influenced areas [40], [41], [42]. OTU 824 was the taxa with the highest relative importance in all the drainage samples and was responsible for the dominance index in this environment. Other researchers have reported the *Meiothermus* phylum, *Deinococcus*/*Thermus*, in river sediments affected by an arsenic mine [16] as well as in acid mine drainage [12], albeit in smaller proportions compared to the present work. This is the first time that the genus has been found in great abundance in mine environments, suggesting a broad adaptive capacity of the *Meiothermus* species.

OTUs 3607 and 4974 were classified as *Gemmatimonas* and were more frequently found in the soil samples. Together, these OTUs accounted for 4.43% of the difference observed between the soil and drainage samples. The genus *Gemmatimonas* has been reported in studies of diversity in mine environments that were either uncontaminated or contaminated with acid mine drainage. Mendez *et al*. [43] used 16S rDNA libraries to study the bacterial composition of the Klondyke mine in the USA. *Gemmatimonadetes* was only found in uncontaminated samples, where the pH was around 8.0. Reis *et al.* [17] studied sediments affected by heavy metals from mining areas in Brazil and found that the phylum *Gemmatimonadetes* was only present in contaminated environments. It is therefore possible that these bacteria were widely present in the soil of the Sossego mine region and resisted the impact of the drainage, although their abundance decreased slightly due to the prevalence of other bacteria more resistant to heavy metals.

The OTU 1366 was identified as *Acidobacteria*, a phylum that includes genera commonly found in soil and drainage environments [14], and OTU 303 was identified only as bacteria present in soils.

The MRT and ABT analyses revealed a major influence of copper on the bacterial community of the Sossego mine. Although neutral pH decreases the available copper, its presence can still interfere with the microbial population. Kunito *et al*. [34] evaluated the influence of different forms of copper on the soil microorganism community, and found no significant correlation between total copper and the tolerance of the bacteria. Nonetheless, in alkaline soils a small (but important) proportion (about 4% of the total copper) can remain adsorbed onto soil particles and/or associated with organic matter. The copper fraction associated with organic matter is slightly soluble, and could have a negative impact on the bacterial community. Furthermore, the soluble and adsorbed fractions of copper are in equilibrium; although the soluble copper reflects the toxicity, the adsorbed copper reflects the toxicity capacity, because it is the source of the soluble copper. In soil with a high content of heavy metals that compete for adsorption sites, copper is the cation most likely to be retained by organic matter, clay, and oxides. This competition process mainly occurs in soil where there are large inputs of limestone, due to the increase in soil sorption capacity [44]. It is therefore possible that copper influenced the structure and composition of the bacterial communities of the Sossego mine, because the soil was exposed to different conditions of rainfall, redox condition, and limestone application.

The most interesting effect of copper was observed for the *Deinococcus*/*Thermus* phylum, which showed a strong positive correlation with this metal, as well as with other metals, such as zinc and nickel (see the Spearman rank correlation coefficients). This phylum includes the genera *Deinococcus*, *Meiothermus*, *Thermus*, and *Trupera*, which have shown resistance to extreme conditions (high irradiation, oxidation, high temperature, and desiccation) [45], [46]. Although they have a broad adaptation capacity, little is known about their survival in the presence of high levels of heavy metals, because (until the present study) this phylum has only been detected at low abundance in mine environments. The ecological importance of *Deinococcus*/*Thermus* was only proposed recently [47], and their presence in mine environments could provide new opportunities for the study of these microorganisms.

Another important factor that affected bacterial composition in the Sossego mine was the potassium concentration. Both potassium and sodium were present at high levels in the soil samples, and were highly inter-correlated (r = 0.94). Both elements could have been influenced by the plant rhizosphere, which was present in most of the soil samples. Potassium is a plant macronutrient that is easily leached because of its mobility in soils. Bacteria can affect the solubility and availability of this and other nutrients, which affects the growth of plants as well as the selection of specific bacteria associated with potassium [48].

The MRT analysis revealed high bacterial diversity in samples with elevated pH. Since there was high exposure to limestone, the bacterial community of the drainage and soil could have become adapted to neutral/alkaline conditions, which would explain the high abundance of genera generally associated with high pH, such as *Gemmatimonas* and *Meiothermus*.

In conclusion, it was found that a chronic impact of neutral mine drainage in the soil environment shifted the composition and structure of the bacterial community, but did not affect bacterial diversity. The high diversity found in both environments could have been due to constant exposure to the drainage, as well as the high availability of organic matter that promoted the growth of an adapted microbial community. The high levels of heavy metals, as well as the concentrations of nutrients such as potassium and sodium, could account for the different microbial compositions of the drainage and soil samples. The *Deinococcus/Thermus* phylum was strongly affected by heavy metals (especially copper), and the *Meiothermus* genus was dominant in the drainage communities. This suggests that the full diversity of these taxa remains unexplored.

## Supporting Information

Figure S1 **Single regression tree.** Showing the relation between standardized diversity indices and chemical parameters. A: Berger-Parker; B: Simpson; C: Shannon.
(DOCX)

Table S1 **Locations and chemical parameters of the drainage and soil samples.** Metal concentrations are expressed in mg/kg. The organic matter (OM) content is expressed in g/dm^3^.
(DOCX)

Table S2 **Sample sequencing and diversity index information.**
(DOCX)

Table S3 **Relative influence (%) of chemical parameters in phylum abundance determined by aggregated boosted tree analysis.**
(DOCX)

Table S4 **Spearman rank correlation results.**
(DOCX)
