# Supplementary material for: Changes in the Bacterial Community of Soil from a Neutral Mine Drainage Channel
Source: PLoS One. 2014 May 5;9(5):e96605. doi: 10.1371/journal.pone.0096605 (PMC4010462; doi:10.1371/journal.pone.0096605)
Supplement: Figure S1 — Single regression tree. Showing the relation between standardized diversity indices and chemical parameters. A: Berger-Parker; B: Simpson; C: Shannon. (DOCX) [file pone.0096605.s001.docx]

**
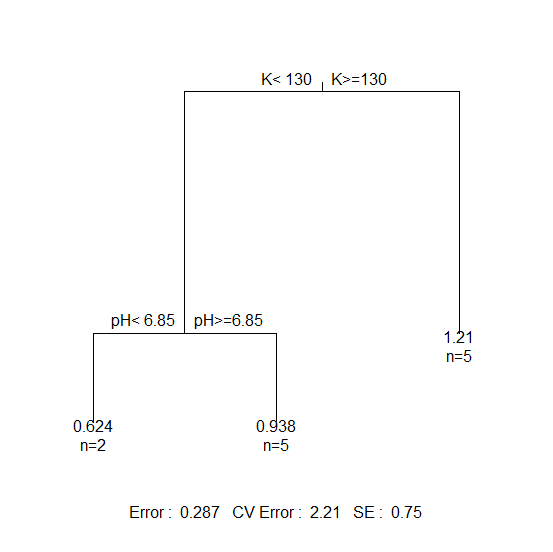

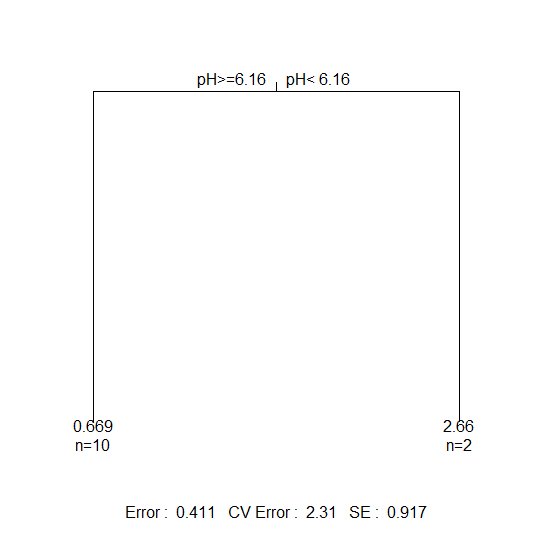

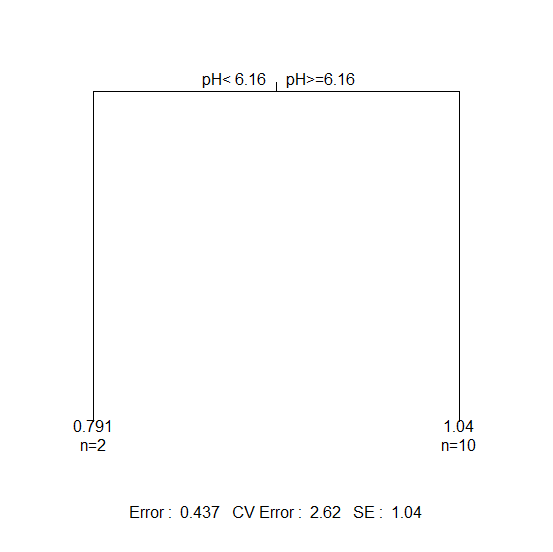
Figure S1**. Single Regression Tree of the relation between standardized diversity indices with chemical parameters. A: Berger-Parker; B: Simpson; C: Shannon.

C

B

A of the relation between standardized diversity indices with chemical parameters
