# Supplementary material for: Changes in the Bacterial Community of Soil from a Neutral Mine Drainage Channel
Source: PLoS One. 2014 May 5;9(5):e96605. doi: 10.1371/journal.pone.0096605 (PMC4010462; doi:10.1371/journal.pone.0096605)
Supplement: Table S1 — Locations and chemical parameters of the drainage and soil samples. Metal concentrations are expressed in mg/kg. The organic matter (OM) content is expressed in g/dm3. (DOCX) [file pone.0096605.s002.docx]

**Table S1**. Localization and chemical parameters of the drainage and soil samples. Metal concentrations are expressed in mg/kg. The organic matter (OM) content is expressed in g/dm^3^

| **Sample** | **Localization** | **Cadmium** | **Calcium** | **Lead** | **Copper** | **Chrome** | **Sulfur** | **Iron** |  |
| --- | --- | --- | --- | --- | --- | --- | --- | --- | --- |
| **D1** | 6 25 35.52S, 50 3 5.66W | 0.8 | 15000 | 5.3 | 27334 | 33.2 | 3000 | 40906 |  |
| **D2** | 6 25 36.06S, 50 3 5.48W | 0.4 | 20300 | 3.2 | 45713 | 9.1 | 1600 | 7825 |  |
| **D3** | 6 25 36.65S, 50 3 5.5W | 0.7 | 5700 | 5 | 38777 | 30.3 | 134000 | 33820 |  |
| **D4** | 6 25 37.32S, 50 3 4.03W | 0.5 | 191000 | 3.2 | 63672 | 11.1 | 5200 | 9834 |  |
| **D5** | 6 25 50.92S, 50 2 58.42W | 0.5 | 8100 | 4.5 | 10234 | 29.1 | 2300 | 30632 |  |
| **D6** | 6 25 57.27S, 50 2 58.86W | 0.7 | 1500 | 10.9 | 2888 | 23.7 | 250 | 42604 |  |
| **S1** | 6 25 35.53S, 50 3 5.8W | 0.4 | 1200 | 3 | 3209 | 15.7 | 280 | 9508 |  |
| **S2** | 6 25 36.09S, 50 3 5.52W | 0.7 | 7900 | 13.2 | 22056 | 43.7 | 171000 | 31834 |  |
| **S3** | 6 25 36.66S, 50 3 5.9W | 0.3 | 131000 | 2.9 | 2120 | 29.7 | 580 | 15997 |  |
| **S4** | 6 25 37.29S, 50 3 4.08W | 0.5 | 6100 | 9.3 | 600 | 27.1 | 430 | 30904 |  |
| **S5** | 6 25 50.94S, 50 2 58.48W | 0.4 | 10500 | 1.7 | 2729 | 23.2 | 490 | 14937 |  |
| **S6** | 6 25 57.28S, 50 2 58.93W | 0.5 | 15100 | 5 | 7009 | 19.3 | 410 | 26995 |  |

Supplementary Table 1. Localization and chemical parameters of the drainage and soil samples. The concentration is expressed in mg/ Kg, exception pH. The organic matter (OM) is expressed in g/dm^3^ (continued)

| **Sample** | **Phosphorus** | **Magnesium** | **Manganese** | **Nickel** | **Potassium** | **Sodium** | **Zinc** | **pH** | **OM** |  |  |
| --- | --- | --- | --- | --- | --- | --- | --- | --- | --- | --- | --- |
| **D1** | 2000 | 14700 | 298 | 135 | 18.6 | 2.8 | 64.2 | 7.79 | 21 |  |  |
| **D2** | 1900 | 6800 | 473 | 147 | 2.3 | 1 | 20.3 | 7.81 | 21 |  |  |
| **D3** | 58500 | 10700 | 206 | 112 | 13.2 | 2.8 | 37.8 | 6.08 | 39 |  |  |
| **D4** | 3600 | 4000 | 515 | 127 | 1.6 | 1 | 20.9 | 7.62 | 25 |  |  |
| **D5** | 1500 | 8500 | 279 | 123 | 18.6 | 2.3 | 30.6 | 8.05 | 13 |  |  |
| **D6** | 220 | 950 | 178 | 29.5 | 1.6 | 1 | 13.2 | 7.62 | 16 |  |  |
| **S1** | 440 | 3300 | 104 | 57.2 | 94 | 70.5 | 12.2 | 4.86 | 6 |  |  |
| **S2** | 1000 | 1300 | 1178 | 97.5 | 166 | 85 | 16.3 | 6.24 | 30 |  |  |
| **S3** | 610 | 14300 | 291 | 45.9 | 355 | 131 | 7.9 | 8.66 | 13 |  |  |
| **S4** | 290 | 1300 | 690 | 37.7 | 229 | 82.6 | 14.7 | 6.62 | 53 |  |  |
| **S5** | 530 | 6100 | 157 | 97.9 | 538 | 181 | 15.9 | 8.78 | 11 |  |  |
| **S6** | 360 | 3000 | 259 | 53.5 | 229 | 64 | 12.3 | 8.33 | 21 |  |  |
