# Supplementary material for: Changes in the Bacterial Community of Soil from a Neutral Mine Drainage Channel
Source: PLoS One. 2014 May 5;9(5):e96605. doi: 10.1371/journal.pone.0096605 (PMC4010462; doi:10.1371/journal.pone.0096605)
Supplement: Table S2 — Sample sequencing and diversity index information. (DOCX) [file pone.0096605.s003.docx]

**Table S2**. Sample sequencing and diversity index information.

| **Sample** | **Number of reads** | **Number of OTUs** | **Good Coverage** | **Shannon (H)** | **Simpson**  **(1-D)** | **Berger-Parker** |
| --- | --- | --- | --- | --- | --- | --- |
| D1 | 9280 | 1098 | 94.14% | 5.38 | 0.9836 | 0.0814 |
| D2 | 10101 | 358 | 98.15% | 1.956 | 0.5592 | 0.658 |
| D3 | 7780 | 489 | 96.74% | 3.716 | 0.9087 | 0.261 |
| D4 | 4730 | 542 | 94.23% | 4.061 | 0.9159 | 0.255 |
| D5 | 12306 | 1316 | 95.08% | 5.651 | 0.9904 | 0.038 |
| D6 | 5684 | 933 | 91.47% | 5.376 | 0.9837 | 0.091 |
| S1 | 14857 | 422 | 99.04% | 4.115 | 0.9542 | 0.174 |
| S2 | 21198 | 1312 | 97.14% | 4.558 | 0.9505 | 0.181 |
| S3 | 3248 | 593 | 91.23% | 4.867 | 0.9604 | 0.172 |
| S4 | 5596 | 1654 | 83.86% | 6.6 | 0.9965 | 0.024 |
| S5 | 3710 | 676 | 91.48% | 5.447 | 0.9868 | 0.085 |
| S6 | 4165 | 1132 | 86.1% | 6.163 | 0.9942 | 0.042 |
