# Supplementary material for: Changes in the Bacterial Community of Soil from a Neutral Mine Drainage Channel
Source: PLoS One. 2014 May 5;9(5):e96605. doi: 10.1371/journal.pone.0096605 (PMC4010462; doi:10.1371/journal.pone.0096605)
Supplement: Table S3 — Relative influence (%) of chemical parameters in phylum abundance determined by aggregated boosted tree analysis. (DOCX) [file pone.0096605.s004.docx]

**Table S3**. Relative influence (%) of chemical parameters in abundance of phylum determined by Aggregated Boosted Tree Analysis.

|  | **Acidobacteria** | **Actinobacteria** | **Gemmatimonadetes** | **Proteobacteria** | **Deinococcus/Thermus** |
| --- | --- | --- | --- | --- | --- |
| Cadmium | 0.13 | 0 | 25.19 | 0 | 1.75 |
| Calcium | 3.83 | 0.47 | 10 | 0 | 1.16 |
| Lead | 4.96 | 1.41 | 0 | 1.51 | 0.12 |
| Copper | 34.16 | 0 | 0 | 59.74 | 62.42 |
| Chrome | 0.66 | 18.02 | 39.25 | 0 | 1.13 |
| Sulfur | 2.23 | 0.44 | 0 | 3.76 | 1.22 |
| Iron | 0 | 0 | 22.44 | 16.24 | 0.93 |
| Phosphorus | 1.35 | 0 | 0 | 0 | 4.75 |
| Magnesium | 0.54 | 1.49 | 0 | 0 | 0.25 |
| Manganese | 0.97 | 0.28 | 0 | 0 | 2.04 |
| Nickel | 0.04 | 8.66 | 0 | 0 | 3.66 |
| Potassium | 44.14 | 62.24 | 0 | 13.58 | 18.65 |
| Sodium | 2.6 | 3.29 | 0 | 3.17 | 1.68 |
| Zinc | 0.38 | 0 | 0 | 0 | 0.16 |
| pH | 3.76 | 3.72 | 0 | 2 | 0.08 |
| Organic Matter | 0.25 | 0 | 0 | 0 | 0 |
