# Supplementary material for: Changes in the Bacterial Community of Soil from a Neutral Mine Drainage Channel
Source: PLoS One. 2014 May 5;9(5):e96605. doi: 10.1371/journal.pone.0096605 (PMC4010462; doi:10.1371/journal.pone.0096605)
Supplement: Table S4 — Spearman rank correlation results. (DOCX) [file pone.0096605.s005.docx]

**Table S4**. Spearman rank correlation results.

|  | Acido | Actino | Gemma | Proteo | Dei/The | Cd | Ca | Pb | Cu | Cr | S | Fe | P | Mg | Ni | Mn | K | Na | pH | Zn | OM |
| --- | --- | --- | --- | --- | --- | --- | --- | --- | --- | --- | --- | --- | --- | --- | --- | --- | --- | --- | --- | --- | --- |
| Acido | 1.00 | 0.69 | 0.19 | 0.62 | -0.34 | 0.18 | -0.34 | 0.31 | -0.34 | 0.45 | -0.04 | 0.22 | -0.31 | -0.20 | -0.26 | 0.15 | 0.45 | 0.50 | -0.25 | -0.18 | -0.10 |
| Actino |  | 1.00 | 0.36 | 0.52 | -0.20 | 0.09 | -0.48 | 0.19 | -0.24 | 0.45 | 0.17 | 0.13 | -0.11 | -0.17 | -0.22 | 0.02 | 0.44 | 0.51 | -0.35 | -0.05 | 0.06 |
| Gemma |  |  | 1.00 | 0.28 | 0.29 | 0.61 | 0.11 | 0.45 | 0.37 | 0.73 | 0.76 | 0.56 | 0.51 | 0.25 | 0.22 | 0.41 | -0.14 | -0.07 | -0.08 | 0.55 | 0.40 |
| Proteo |  |  |  | 1.00 | 0.36 | 0.52 | -0.64 | 0.57 | 0.16 | 0.35 | 0.15 | 0.36 | -0.01 | -0.26 | 0.06 | 0.11 | -0.30 | -0.17 | -0.67 | 0.27 | 0.08 |
| Dei/The |  |  |  |  | 1.00 | 0.46 | -0.04 | 0.28 | 0.85 | 0.02 | 0.62 | 0.08 | 0.67 | 0.11 | 0.67 | 0.25 | -0.74 | -0.59 | -0.44 | 0.70 | 0.39 |
| Cd |  |  |  |  |  | 1.00 | -0.32 | 0.84 | 0.35 | 0.56 | 0.37 | 0.85 | 0.21 | -0.12 | 0.09 | 0.22 | -0.44 | -0.34 | -0.43 | 0.59 | 0.55 |
| Ca |  |  |  |  |  |  | 1.00 | -0.39 | 0.31 | -0.24 | 0.31 | -0.43 | 0.39 | 0.42 | 0.43 | 0.43 | 0.06 | -0.09 | 0.65 | 0.10 | 0.04 |
| Pb |  |  |  |  |  |  |  | 1.00 | 0.08 | 0.49 | 0.15 | 0.79 | -0.16 | -0.47 | -0.24 | 0.42 | -0.29 | -0.22 | -0.46 | 0.24 | 0.64 |
| Cu |  |  |  |  |  |  |  |  | 1.00 | -0.13 | 0.67 | -0.11 | 0.82 | 0.30 | 0.82 | 0.28 | -0.69 | -0.65 | -0.24 | 0.71 | 0.34 |
| Cr |  |  |  |  |  |  |  |  |  | 1.00 | 0.48 | 0.76 | 0.17 | 0.25 | -0.12 | 0.23 | 0.23 | 0.37 | -0.14 | 0.29 | 0.28 |
| S |  |  |  |  |  |  |  |  |  |  | 1.00 | 0.13 | 0.86 | 0.44 | 0.64 | 0.56 | -0.21 | -0.07 | -0.16 | 0.73 | 0.50 |
| Fe |  |  |  |  |  |  |  |  |  |  |  | 1.00 | -0.08 | -0.10 | -0.29 | 0.06 | -0.14 | -0.06 | -0.23 | 0.29 | 0.38 |
| P |  |  |  |  |  |  |  |  |  |  |  |  | 1.00 | 0.70 | 0.85 | 0.28 | -0.40 | -0.32 | -0.10 | 0.78 | 0.28 |
| Mg |  |  |  |  |  |  |  |  |  |  |  |  |  | 1.00 | 0.60 | -0.06 | 0.03 | 0.00 | 0.34 | 0.45 | -0.18 |
| Ni |  |  |  |  |  |  |  |  |  |  |  |  |  |  | 1.00 | 0.22 | -0.39 | -0.41 | 0.08 | 0.78 | 0.07 |
| Mn |  |  |  |  |  |  |  |  |  |  |  |  |  |  |  | 1.00 | -0.08 | -0.07 | -0.09 | 0.29 | 0.69 |
| K |  |  |  |  |  |  |  |  |  |  |  |  |  |  |  |  | 1.00 | 0.94 | 0.41 | -0.49 | -0.24 |
| Na |  |  |  |  |  |  |  |  |  |  |  |  |  |  |  |  |  |  | 0.18 | -0.45 | -0.17 |
| pH |  |  |  |  |  |  |  |  |  |  |  |  |  |  |  |  |  |  | 1.00 | -0.14 | -0.42 |
| Zn |  |  |  |  |  |  |  |  |  |  |  |  |  |  |  |  |  |  |  | 1.00 | 0.41 |
| OM |  |  |  |  |  |  |  |  |  |  |  |  |  |  |  |  |  |  |  |  | 1.00 |

Acido: *Acidobacteria*; Actino: *Actinobacteria*; Gemma: *Gemmatimonadetes*; Proteo: *Proteobacteria*; Dei/The: *Deinococcus/Thermus*
